# Resilience Within Families of Young Children with ASD

**DOI:** 10.1007/s10803-025-06773-4

**Published:** 2025-03-06

**Authors:** Leanne Dijkstra-de Neijs, Hanna Swaab, Ina A. van Berckelaer-Onnes, Wietske A. Ester

**Affiliations:** 1 Sarr Autism Rotterdam, Youz Child- and Adolescent Psychiatry, Parnassia Group, Dynamostraat 18, 3083 AK Rotterdam, The Netherlands; 2 Clinical Neurodevelopmental Sciences, Leiden University, Leiden, The Netherlands; 3 Parnassia Group, Parnassia Academy, Den Haag, The Netherlands; 4 Curium-LUMC, Department of Child- and Adolescent Psychiatry, Leiden University, Oegstgeest, The Netherlands; 5 Leiden Institute for Brain and Cognition, Leiden University, Leiden, The Netherlands

**Keywords:** Resilience within families, Parenting stress, Autism spectrum disorder, Young children

## Abstract

Resilience within families may temper the risk of high parenting stress faced by parents of young children with ASD. Within families, individual differences between parents may contribute differently to resilience. There is a lack in knowledge regarding the contribution of intrapersonal and contextual factors associated with resilience in parents of young children with ASD within the same family. In this cross-sectional study (n=249 individuals), resilience within families is addressed by investigating (1) family parenting stress, (2) associated factors contributing to maternal (n=87) and paternal (n=74) resilience, and (3) relating to resilience within families (n=74) of 3-to-6-year-old children with ASD (n=88). (1) The percentage of families with regular parenting stress in both parents (33%) is almost equal to the proportion of families with (sub)clinical parenting stress in both parents (36%), families with mothers experiencing (sub)clinical and fathers experience regular parenting stress are twice as common (22%) than the other way around (9%). Contributors to (2) mothers’ resilience to parenting stress are good planning/organizing skills and satisfactory social relations. Contributors to fathers’ resilience are low levels of ‘worrying’ and good social relations. The shared contributing factor to resilience within families (3) is the satisfaction of both parents with their social relations. Most of the parents of the same young child with ASD experience a comparable degree of parental stress, with different dynamics in individual parents contributing to resilience within families. This suggests the need for a personalized parental approach in families with young children with ASD.

## Introduction

Resilience within families may reduce the risk of chronic parenting stress that can have major psychological and physical consequences in parents of young children with ASD and that may influence family functioning (Fairthorne et al., 2014; Hayes & Watson, 2013; Rezendes & Scarpa, 2011; Riahi & Izadi-Mazidi, 2012; van der Lubbe et al., 2022; Zhou et al., 2019). Understanding differences and dynamics between parents in coping with parental stress within families is important to understand resilience within families in challenging parenting situations and may reveal pathways to optimize family support for families with young children with ASD (Benson & Karlof, 2009). Therefore, it is essential to learn more about factors that contribute to resilience in parents to parenting stress. So far, little is known about resilience in fathers and in parents within the same family of young children with ASD, as most studies report on mothers of school-aged and/or older children at a group level (Bekhet et al., 2012; Ilias et al., 2018).

### Resilience

Resilience can be defined as the individual ability to quickly recover after an unpleasant incident, like a stressful parenting situation, it is a multifaceted construct, influenced by various biological, psychological, and social factors (Bateman et al., 2005; Tugade & Fredrickson, 2004). The concept of family resilience transcends the perspective of viewing individual family members as potential resources for personal resilience to parenting stress (Walsh, 2003). It emphasizes the examination of resilience within the family as a cohesive functional unit (Walsh, 2003). Individual- and family resilience are therefore particularly broad but different concepts, where using a framework such as the family resilience framework by Walsh (2003) can clarify. Given the gap in knowledge on individual and family factors contributing to resilience to parenting stress in young children with ASD, especially in fathers, separate research into factors that contribute to fathers' and mothers' resilience could be a first step after which resilience within families of the same young child can be explored.

### Intrapersonal Factors and Resilience

Intrapersonal factors are the personal characteristics and factors of an individual that influence their actions, beliefs, and thoughts that contribute to their overall health and well-being (Harley et al., 2020). While there is robust literature on intrapersonal factors that can contribute to resilience (Joyce et al., 2018; Wu et al., 2013), and growing insight in resilience to parenting stress (Fang et al., 2024), when it comes to resilience to parenting stress in mothers and fathers of young children with ASD, studies are more limited (Bekhet et al., 2012; Ilias et al., 2018). Current literature indicates that mothers with good parenting efficacy, i.e., feelings of competency in their parental role, have been associated with well-being and positive parenting outcomes (Kuhn & Carter, 2006). Parenting efficacy was also positively associated to mother’s locus of control, i.e., the sense of control that mothers feel in their life/their capacity to influence their own thoughts and behavior and to be actively engaged with their child (Kuhn & Carter, 2006). The lack of parenting efficacy as well as maternal anger related to depression over time in mothers of toddlers with ASD(Carter et al., 2009; Ilias et al., 2018). Additionally, a low sense of coherence, reflecting parents’ view on life and capacity to respond to stressful situations, contributed to negative coping strategies in parents’, mostly in mothers of 3- to 7-year-olds with ASD (Pisula & Kossakowska, 2010; Siah & Tan, 2015). Also, frequent maternal anger, anxiety and worrying are associated to symptom severity in the child, stress proliferation, and increased negative thoughts in mothers of children with ASD aged 6–9-year-olds (Benson & Karlof, 2009). In summary, intrapersonal factors related to resilience to parenting stress in parents, mostly known in mothers of children with varying ages with ASD are parenting efficacy, locus of control, regulation of emotions and negative thoughts/worrying (Bekhet et al., 2012; Ilias et al., 2018).

### Contextual Factors and Resilience

Known contextual factors that contribute to parental resilience are the presence of social support, financial health, child’s age and ASD severity. Social support refers to the parents’ perception that they can be helped or understood by a close person (Siman-Tov & Kaniel, 2011). Social support, in most studies from family, friends or a significant other, has been identified as a protective factor in parenting stress in several studies, mostly in mothers of children with ASD in broad age ranges (2 till 21 years) (Ekas & Lickenbrock, 2010; Li et al., 2022; Siman-Tov & Kaniel, 2011; Tobing & Glenwick, 2007; Weinberg et al., 2021). Social support related to increased optimism, less stress, and fewer negative thoughts in mothers of children with ASD younger than 18 years (Ekas & Lickenbrock, 2010). Financial health was also a factor that was frequently reported to influence the level of stress among parents of children with ASD (Trentacosta et al., 2018). Also, the age of the child and ASD severity appear to be important, wherein parents with older children and lower ASD severity reported less parenting stress (McStay et al., 2013). Moreover, Kotera et al. (2020) emphasized the role of resilience within families as a contributing factor to the better coping capability of parents of children with ASD, suggesting that interventions aiming at enhancing resilience within families may positively impact parental well-being including reduced parenting stress (Kotera et al., 2020).

### Parenting Stress

Parenting stress refers to the tension and pressure that parents experience while raising their child (Vermulst et al., 2015). Although a certain degree of parenting stress is desirable, chronic and/or overwhelming parenting stress may shift from a balanced mental and physical equilibrium, in which stress has a benefit of adaptation, toward distress: a feeling of extreme worry, sadness, or pain (Dijkstra-de Neijs et. al., 2020).

The importance of enhancing resilience within families to parenting stress is illustrated by the finding that mothers of children with ASD experience 1.5-times higher levels of daily parenting stress and that they present significant levels of stress-related mental and/or physical problems with elevated mortality hazard ratios compared to mothers of typically developing children (Dijkstra-de Neijs et al., 2020; Estes et al., 2009; Rezendes & Scarpa, 2011; Riahi & Izadi-Mazidi, 2012). Studies on paternal parenting stress reported varying results (Baker- Ericzén et al., 2005; Flippin & Crais, 2011; Grebe et al., 2021; Pisula & Porębowicz-Dörsmann, 2017; Rivard et al., 2014; Soltanifar et al., 2015). Relevant is that parenting stress in mothers and fathers seem to be different. Studies exploring the differences between parenting stress in fathers and mothers found varying results: some studies reported that fathers and mothers both experience high levels of parenting stress, others report that stress was only present in mothers (Baker-Ericzén et al., 2005; Flippin & Crais, 2011; Grebe et al., 2021; Pisula & Porębowicz-Dörsmann, 2017; Rivard et al., 2014; Soltanifar et al., 2015). Results within studies on the origins of parenting stress also vary, some studies show that both fathers and mothers experience the most parenting stress from the parent–child relationship, while other studies display that mothers experience more stress from their parenting role than fathers (Davis & Carter, 2008; Dijkstra-De Neijs et al., 2024). It was also reported that effects of parenting stress may affect mothers and fathers in different ways. Maternal parenting stress was related to disinhibited eating, abdominal obesity and metabolic syndrome. However, this association was not present in fathers (van der Lubbe et al., 2022).

The high risk for parenting stress among parents of young children with ASD, emphasizes the need consider parenting stress as a pathway to mental and physical health of both parents and the child. There is a need to understand parental resilience to stress in the context of raising a child with ASD. Given the limited literature available on stress and resilience to stress in fathers and mothers of young children with ASD, especially within the same family, the present study investigates family parenting stress i.e., do both parents within the same family with a young child with ASD experience parenting stress. In addition, we explore associated intrapersonal and contextual factors with fathers and mothers’ resilience.

## Methods

### Study Design

The present study is a descriptive, correlational, cross-sectional study, assessing parental resilience in parents of young children with ASD. This study is part of the ongoing Tandem study (Dutch trial register: NL7534), approved by the Institutional Review Board of Leiden University Medical Center, the Netherlands (NL6378.058.18).

### Participants

Parents were eligible for inclusion if 1) their child was diagnosed with ASD, and 2) the child was aged 3 to 7 years. Parents were recruited from Youz Parnassia Group, GGZ Delfland, and Yonx all mental health care providers in the Netherlands. Parents eligible for inclusion were asked by their child's psychologist for consent to be contacted by the research team shortly after their child being diagnosed with ASD. If parents agreed, contact details of the parents were passed on to the research team. The research team then screened the parents by telephone to verify whether they met the inclusion criteria and if they were willing to participate. If they did, an informed consent meeting took place where parents received detailed information about their study participation and signed an informed consent form. After informed consent, a research employee provided parents with a link with which they could enter explanation about the study and complete their questionnaires within Research Manager, a data management program. If parents had questions about the questionnaires, they could contact an employee of the research team by mail or telephone.

### Measures

*Parenting stress* was assessed by self-report with the total parental stress scale from the Dutch parenting burden questionnaire (In Dutch: *Opvoedbelasting Vragenlijst* [OBVL] (Vermulst et al., 2015). The OBVL comprises out of 34 questions and uses a 1–4 response scale (1 = does not apply, 2 = does somewhat apply, 3 = does apply, 4 = totally does apply). Raw scores, used within the statistical analysis within this study, can be transformed into T-scores which give clinical meaning using norm tables, as presented by Vermulst et al. (2015). T-scores below 60 are seen in parents with a degree of desirable parenting stress, referred to in this article as ‘regular parenting’ stress. Scores of 60 < are interpreted as parents with moderate to severe parenting stress, which requires attention for the well-being of both parent and child and will be referred to in this article as ‘(sub)clinical parenting stress’. Explorative factor analysis has shown that the total parental stress scale within the OBVL is empirically demonstrated (Vermulst et al., 2015). The results of the factor analysis show that there is a good fit, which means that the theoretically assumed scales are also empirically confirmed (CFI = 0.965, RMSEA = 0.045) (Veerman et al., 2014; Vermulst et al., 2015). The norm population used within this study is the OBVL norm population (Vermulst et al., 2015). The norm population included data on 848 mothers of typically developing Dutch children (50.8% boys) from the general population (Vermulst et al., 2015). Within the literature it is common to use the Parental Stress Index (PSI) to operationalize parenting stress, with no Dutch version available we chose the OBVL, as the OBVL focuses mainly on characteristics of the parent in relation to upbringing and the quality of the parent–child relationship. This can be compared with the parent domain of the PSI (Abidin, 1983; Veerman et al., 2014; Vermulst et al., 2015).

### Operationalizing Resilience

**Fig. 1:**
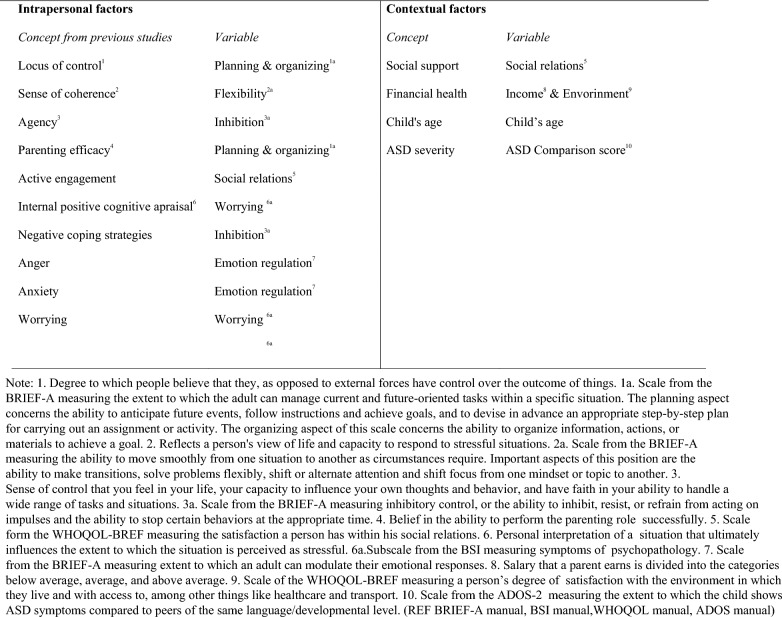
Operationalization of factors that contribute to resilience to parenting stress in families with a young child with ASD

To gain insight into factors that may contribute to resilience to parenting stress, we searched the literature with help of the search terms: ‘parenting stress', ‘parenting resilience', 'ASD', and resilience within families in the search engines PubMed and Google scholar with no time limit. The most recent peer-reviewed studies on resilience to parental stress are described within this study to gain insight into the current state of knowledge on this subject. To operationalize the factors, as found in our literature search, that contribute to resilience to parenting stress, we matched these factors to corresponding measuring instruments. For example, the factor 'locus of control' i.e., the degree to which people believe that they, as opposed to external forces have control over the outcome of things, was operationalized through the planning and organizing scale from the BRIEF-A. This scale measures the extent to which a person can manage current and future-oriented tasks, can anticipate to future events, can follow instructions, and achieve goals. The operationalized resilience factors are shown in Fig. 1. The corresponding measurement variables are further described below. To minimize the risk on a type II error we considered the research design by only adding variables from previous studies in line with our study i.e., studies on parents of children with ASD of older age and operationalizing them, which is visualized within Fig. 1, and by adding important covariates from previous literature that may influence errors within our analyses, such as ASD severity of the child. Subsequently we examined interaction effects and curvilinearity.

### Intrapersonal Factors

Parental planning & organizing, inhibition, and flexibility are subscales from the Behavior Rating Inventory of Executive Function-Adult questionnaire informant list (BRIEF-A) that measures an adult’s executive functioning at a descriptive behavioral level and is filled in by the other parent/partner, or a close acquaintance (Roth et al., 2011). Although the BRIEF-A is a questionnaire developed for screening executive functions in adults, at item level this is done by assessing daily behavior, i.e., adaptive functioning which emerge from a person’s underlying executive functions. The *Planning & organizing scale* measures the extent to which the adult can manage current and future-oriented tasks within a specific situation. The planning aspect concerns the ability to anticipate future events, follow instructions and achieve goals, and to devise in advance an appropriate step-by-step plan for carrying out an assignment or activity. The organizing aspect of this scale concerns the ability to organize information, actions, or materials to achieve a goal. The *Inhibition scale* measures the adult's inhibitory control, or the ability to inhibit, resist, or refrain from acting on impulses and the ability to stop certain behaviors at the appropriate time. The *Flexibility scale* measures the adult's ability to move smoothly from one situation, activity, or issue to another as circumstances require. Important aspects of this position are the ability to make transitions, solve problems flexibly, shift or alternate attention and shift focus from one mindset or topic to another (Roth et al., 2011). Exploratory factor analysis showed that executive functioning within the BRIEF-A can be demonstrated empirically, and the concept validity is good (Roth et al., 2011). The norm population used in this study is the Dutch BRIEF-A norm population, comprising 537 Dutch adults from the general population, age range 18–65 years (Roth et al., 2011). The raw scores on the above-mentioned scales were used in the analyses within this study.

The *Emotion regulation scale* from the BRIEF-A informant list concerns the manifestation of executive functions in the domain of emotions. This scale measures the extent to which an adult can modulate emotional responses. Weak emotion regulation can manifest itself in emotional lability or emotional explosiveness. To operationalize parental worrying, depressive thoughts, and internal positive cognitive appraisal the Depressive mood subscale from the Brief Symptom Inventory (BSI) was used (De Beurs, 2008) (Fig. 1). In this study the depressive mood subscale will be referred to as ‘worrying’, to clarify on whether there is actual psychopathology such as having a depression or parents are more worried/ruminate more on negative thoughts than other parents, the latter being what the depression subscale of the BSI measures. We find that worrying falls under the intrapersonal factors, as excessive worrying is related to gloominess or even depression which in turn is associated to maternal anger (Carter et al., 2009). The BSI is a self-report questionnaire which that provide insight into both psychopathological and psychological symptoms (De Beurs, 2008). Each item on the BSI is rated on a 5-point Likert scale, ranging from 0 ("not at all") to 4 ("extremely"). The BSI has been shown to have robust psychometric properties, with internal consistency coefficients ranging from 0.71 to 0.85 in its original administration. The norm population used in this study is the BSI norm population, comprising 1037 non-clinical adults above 18 years (Derogatis, 1993). The raw scores on the above-mentioned scales were used in the analyses within this study.

### Contextual Factors

*Social support* and *social economic status (SES)* are assessed using parental self-report on the 'Social relationships’ and ‘Environment’ scale of the World Health Organization Quality of Life (abridged version) questionnaire (WHOQoL-BREF), (World Health Organization, 1996). The WHOQoL-BREF is a self-report questionnaire comprising 26 items with a 1–5 response scale (1 = not at all, 2 = almost not, 3 = average, 4 = pretty good, and 5 = completely). Internal consistency, item–total correlations, discriminant validity, and construct validity through confirmatory factor analysis indicate good to excellent psychometric properties of reliability and tests of validity (Skevington et al., 2004; Trompenaars et al., 2005). The social support scale comprises out of three questions on satisfaction with personal relations, sex life and social support from friends. The environment scale comprises eight questions on how satisfied a person is with the environment they live in. Literature recommends that normative values obtained in the general population serve as norm population (Fayers & Machin, 2007). Since Dutch general population norms are lacking, a cohort from the general Australian population (n = 396, 64% males, mean age of 48.2 years, *sd* = 17.3) assessed by Hawthorne et al. (Hawthorne et al., 2006) serves as norm group.

*Financial health* was retrieved from a self-reported social-demographic questionnaire. In which parents could indicate within which income class they fall, namely: below average/less than 2800 euros per month, average/around 2800 euros per month, above average/more than 2800 euros per month, based on 2017 data from Statistics Netherlands (CBS).

*ASD severity* was assessed by the ADOS-2 comparison score which reflects the degree of severity of the child's ASD symptoms compared to children of the same age. The ADOS-2 is a semi-structured, clinical observation instrument designed to provide a standardized measurement of ASD symptoms in children and adults (De Bildt, A., de Jonge, M., Lord, C., Rutter, M., DiLavore, P., Risi, 2014). It comprises 17 observational items which lead to a total score between 0–46. It has two subscales: (1) the social affect scale, and (2) the restricted and/or repetitive behavior scale. Items can be scored from 0 (typical behavior) to 2 (atypical behavior). We used modules 1, 2 and 3 in our study, depending on the child’s level of speech (module 1: cut off if child speaks few to no words: 11, if child speaks few words: 8. Module 2: cut-off if younger than 5 years: 7, if older than 5 years: 8. Module 3: cut-off = 7) (De Bildt, A., de Jonge, M., Lord, C., Rutter, M., DiLavore, P., Risi, 2014). The interclass correlations for the social affect domain, the restrictive and/or repetitive behavior domain, and the total score were all high, varying from 0.68 to 0.98, in modules 1, 2 and 3. Exploratory factor analysis showed a high conceptual and predictive validity for the modules we used in this study (De Bildt, A., de Jonge, M., Lord, C., Rutter, M., DiLavore, P., Risi, 2014). All ADOS-2 measurements in this study were conducted and scored by certified ADOS-2 practitioners.

*Age of the child* was checked and filled out by the researcher who included the child and their parents within this study.

### Statistical Analysis

We used a cross-sectional study design to gain insight into factors contributing to resilience to parenting stress in parents of young children with ASD. We explored if the operationalized variables (Fig. 1) were normally distributed and were then compared with norm populations using the Chi square test.

To study the presence of family parenting stress i.e., do both parents in families with a young child with ASD experience parenting stress, we calculated percentages of families in which both parents experienced regular or (sub)clinical parenting stress, and in which family’s fathers reported regular, and mothers reported (sub)clinical, or fathers reported (sub)clinical and mothers reported regular parenting stress, measured with the T-scores from the OBVL total scale.

To study associated factors with maternal and paternal resilience, correlations were used. Variables that were significantly correlated with maternal or paternal parenting stress were included in two linear regression analyses using the enter method: one for fathers and one for mothers. Herein data from single/solo participating parents were included.

To explore which of these individual parental resilience factors relate to family parenting stress, a family parenting stress variable was created. The family parenting stress variable comprises the added raw scores of maternal and paternal parenting stress, measured with the OBVL total score, within the same family, or parents of the same young child with ASD. Herein data from single/solo participating parents were excluded. Variables that significantly relate to maternal or paternal parenting stress from the previous linear regression analysis are included as independent variables in a third regression analysis with family parenting stress as the dependent variable, when assumptions where met. Within this linear regression, we also examined whether, in addition to main effects, there could be interaction effects or curvilinearity. To examine interaction effects interaction variables were created by multiplying the independent variables with each other. A higher arc multiple regression analysis was than performed entering the independent variables in step one and the interaction variables in step two. Curvilinearity was examined by creating squared variables of the independent variables. A higher arc multiple regression analysis was than performed entering the independent variables in step one and the squared independent variables in step two.

## Results

### Descriptives

A total of 249 individuals participated within this study comprising 74 families with data from both mother (n = 87) and father (n = 78) of the same child with ASD (n = 88, 80.4% boys). Although welcomed, within this study population there were no families present with other family makeups than single parent or one father and one mother. There were 13 single/solo mothers and 1 single/solo father. Mothers of young children with ASD had higher levels of parenting stress than mothers of typically developing children (*χ*^*2*^*(2)* = 85.7*, p* < 0.01). Mothers also showed more difficulties with their inhibition skills (*χ*^*2*^*(2)* = 13.8*, p* < 0.01) and experienced a poorer quality of their social relations (*χ*^*2*^*(2)* = 273.4*, p* < 0.01) and social economic status (*χ*^*2*^*(2)* = 106.9*, p* < 0.01) than women from the norm population. Additionally, mothers reported earning less compared to the Dutch female population (*χ*^*2*^*(2)* = 6.5*, p* < 0.05). Fathers were between 25 and 58 years old (Mean = 38.2, *sd* = 7.5). Fathers experience higher levels of parenting stress than mothers of typically developing children (*χ*^*2*^*(2)* = 141.1*, p* < 0.01). Fathers experienced a poorer quality on their social relations (*χ*^*2*^*(2)* = 124.8*, p* < 0.01) and social economic status (*χ*^*2*^*(2)* = 150.2*, p* < 0.01) than males from the norm population. Fathers reported earning more compared to the Dutch male population (*χ*^*2*^*(2)* = 12.48*, p* < 0.01) (Table 1). Children were aged between 3 and 7 years old (Median = 5, IQR = 2). Autism severity (ADOS-2) scores ranged from 1 (low ASD severity) to 10 (high ASD severity) (Median = 8, IQR = 3).

**Table 1:** Descriptives of the participating mothers and fathers of young children with ASD within this study compared to the norm population

	Mothers of child with ASD	ASD mothers vs. comparison group		*Chi-square*	*p*	Fathers of child with ASD	ASD fathers vs. comparison group		*Chi-square*	*p*	Comparison group
	N	%	Expected %			N	%	Expected %			
**Parenting stress**											
(Sub)clinical	87	42.5	16.0	85.7	< .01	74	56.8	16.0	141.1	< .01	OBVL norm population (n = 847) (Vermulst et al., 2015)
**Intrapersonal factors**											
Inhibition											BRIEF-A informant list norm population (n = 1082) (Roth et al., 2011)
(Sub)clinical	89	20.4	12.0	13.8	< .01	87	17.2	12.0	2.0	Ns	
Flexibility											
(Sub)clinical	84	16.7	15.0	.16	Ns	72	13.9	15.0	.07	Ns	
Planning & organizing											
(Sub)clinical	84	20.2	16.0	1.03	Ns	72	8.3	16.0	3.01	Ns	
Emotion regulation											
(Sub)clinical	84	12.9	13.0	.02	Ns	72	19.4	13.0	1.7	Ns	
Worrying											BSI norm population (n = 1037) (Derogatis, 1993)
(Sub)clinical	87	10.4	8.8	8.4	Ns	74	12.8	8.8	11.1	Ns	
**Contextual factors**											
Social relationships											Whoqol-Bref norm population (n = 376 males and n = 481 females) (Hawthorne et al., 2006)
(Sub)clinical	87	18.1	5.2	273.4	< .01	74	33.3	10.1	124.8	< .01	
Satisfaction with SES											
(Sub)clinical	83	56.6	5.2	106.9	< .01	74	26.4	10.1	150.2	< .01	
Financial health											Dutch female population (n = 684,940) and Dutch male population (n = 633,830) in 2020 (Central Bureau for Statistics Netherlands (https://www.cbs.nl))
Below average	42	60.0	47.4	6.5	< .05	15	21,4	28.8	12.48	< .01	
Average	16	29.8	34.2			15	21,4	34.3			
Above average	19	22,9	18.4			40	57,1	36.7			

Social economic status is measured using the variable 'environment' from the Whoqol-Bref, comprising questions on how a person rates their satisfaction with their living surroundings and accessibility to health care or services. Financial health was retrieved from a self-reported social-demographic questionnaire. In which parents could indicate within which income class they fall, namely: below average/less than 2800 euros per month, average/around 2800 euros per month, above average/more than 2800 euros per month, based on 2017 data from Statistics Netherlands (CBS)

### Family Parenting Stress

**Fig. 2:**
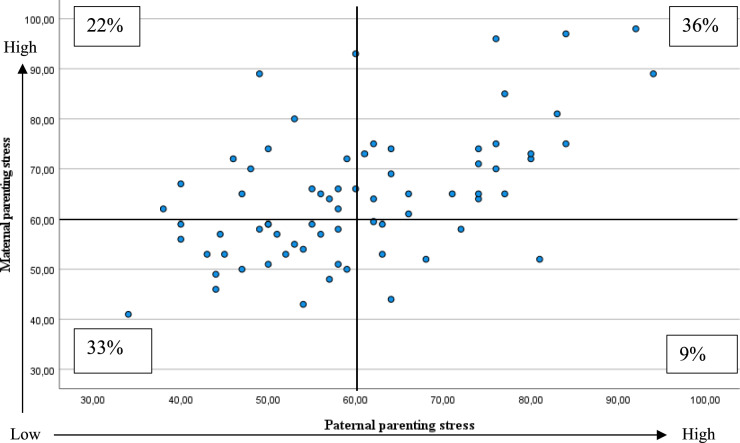
The dynamics between maternal and paternal parenting stress within the same family of a young child with ASD

Of the 74 families in this study, in 32% of the families (n = 24) both mothers and fathers experienced regular parenting stress. In 36% of the families (n = 27) both mothers and fathers experienced (sub)clinical parenting stress. In 22% of the families (n = 16) fathers had regular and mothers (sub)clinical parenting stress an in 9% of the families (n = 7) fathers experienced (sub)clinical and mothers regular parenting stress compared to mothers of typically developing children within the same age group (Vermulst et al., 2015) (Fig. 2).

### Correlations of Paternal and Maternal Resilience Factors and Parental Stress

Mothers with less impulsivity (r = 0.24, p ≤ 0.05), better flexibility (r = 0.35, p ≤ 0.01), planning and organizing skills (r = 0.37, p ≤ 0.01), emotion regulation (r = 0.34, p ≤ 0.01) and less worrying (r = 0.31, p ≤ 0.01) related to lower maternal parenting stress. Mothers’ greater satisfaction with their social relationships (*r* = -0.42, *p* ≤ 0.01) and their social economic status (*r* = -0.26, *p* ≤ 0.05) related to lower maternal parenting stress.

Fathers with better emotion regulation (*r* = 0.27, *p* ≤ 0.05) and less worrying (*r* = 0.48, *p* ≤ 0.01) related to lower paternal parenting stress. Correlations were also found between the degree of fathers’ satisfaction with their social relationships (*r* = -0.52, *p* = < 0.01) and their social economic status (*r* = − 0.44, *p* ≤ 0.01) and paternal parenting stress (Table 2).

**Table 2:** Correlations of mothers (n = 83) and fathers (n = 78) resilience factors and parenting stress (Spearman’s rho)

	Maternal parenting stress	Paternal parenting stress
Inhibition	.24*	.19
Flexibility	.35**	.13
Planning & organizing	.37**	.21
Emotion regulation	.34**	.27*
Worrying	.31**	.48**
Social relationships	– .42**	– .52**
Social economic status	– .26*	– .44**
Financial health	– .08	– .07
ASD severity child	– .08	– .12
Age child	– .10	.00

Higher scores on the inhibition, flexibility, planning & organizing, emotion regulation, depressive mood, ASD severity and maternal/paternal parenting stress means that more problems are experienced in this area. Higher scores on the social relationship, social economic status and financial health indicate that there are less problems within this area

*Correlation is significant at the .05 level (2-tailed)

**Correlation is significant at the .01 level (2-tailed)

### Associations of Parental Stress by Resilience Factors

#### Maternal Parenting Stress

In the regression analysis the enter method was used wherein the variables inhibition, emotion regulation, flexibility, planning & organizing skills, depressive mood, and satisfaction with mother’s social relations and social economic status was entered. Results show that 40% of the variance in maternal parenting stress could be explained by mothers’ planning & organizing skills (F(8,73) = 6.12, *p* = 0.04) and satisfaction with her social relations (F(8,73) = 6.12, *p* ≤ 0.01) (Table 3). *Paternal parenting stress.* In the regression analysis the enter method was used wherein the variables emotion regulation, depressive mood, and satisfaction with fathers’ social relations and social economic status was entered. Results show that 46% of the variance in paternal parenting stress could be explained by fathers’ worrying (F(5,61) = 10.46, *p* ≤ 0.01) and satisfaction with his social relations (F(5,61) = 6.12, *p* = 0.02).

**Table 3:** Regression analysis to identify mothers’ and fathers’ resilience factors by parenting stress measured with the OBVL

		R	R^2^	Adjusted R^2^	SE of the estimate	R^2^ change	Change statistics			
							F change	df1	df2	Sig. F change
	Mothers parenting stress + resilience factors^a^	.63	.40	.33	11.31	.40	6.11	8	73	< .01
	Fathers parenting stress + resilience factors^b^	.67	.46	.41	11.57	.46	10.46	5	61	< .01

	Unstandardized coefficients		Standardized coefficients	t	Sig	95.0% Confidence Interval for B	
	B	SE	Beta			Lower bound	Upper bound
Mothers							
(Constant)	83.12	11.82		7.02	< .01	59.55	106.70
Inhibition	− .66	.55	− .17	− 1.18	.23	− 1.77	.45
Emotion regulation	.06	.41	.02	.14	.88	− .76	.88
Flexibility	.57	.67	.13	.84	.40	− .77	1.91
Planning & organizing skills	.92	.46	.33	2.01	.04	.00	1.84
Worrying	− .26	.32	− .11	− .81	.41	− .91	.38
Social relations	− 1.93	.70	− .36	− 2.73	< .01	− 3.33	− .52
Social economic status	− .37	.73	− .06	− .51	.60	− 1.83	1.08
Fathers							
(Constant)	79.28	13.96		5.67	< .01	51.36	107.21
Emotion regulation	.37	.29	.12	1.27	.20	− .21	.96
Worrying	1.43	.50	.46	2.86	< .01	.43	2.43
Social relations	− 1.52	.63	− .31	− 2.39	.02	− 2.79	− .25
Social economic status	− .60	.90	− .08	− .66	.50	− 2.41	1.20

Dependent variable: maternal parenting stress and paternal parenting stress

^a^Predictors: (constant), social economic status satisfaction, planning & organizing skills, emotion regulation, social relations, worrying, inhibition, and flexibility

^b^Predictors: (constant), social economic status satisfaction, emotion regulation, social relations, worrying

### Associations of Family Parenting Stress by Paternal and Maternal Resilience Factors

The variables that contributed to parenting stress from the previous linear regression analyses for mothers and fathers all correlate with family parenting stress; fathers’ worrying (*r* = 0.36, *p* ≤ 0.01) and satisfaction with his social relations (*r* = 0.55, *p* ≤ 0.01), mothers’ panning & organizing skills (*r* = 0.30, *p* = 0.01) and satisfaction with her social relations (*r* = 0.48, *p* ≤ 0.01). In the family parenting stress regression analysis, the enter method was used wherein the variables fathers’ depressive mood, fathers’ satisfaction with his social relations, mothers’ panning & organizing skills, and mothers’ satisfaction with her social relations, were entered. Results show that 49% of the variance in family parenting stress could be explained by fathers’ and mothers’ satisfaction with their social relations (fathers: F(5,61) = 11.88, *p* = 0.05; mothers: F(5,61) = 11.88, *p* ≤ 0.01). There is no interaction effect or curvilinearity (Table 4).

**Table 4:** Regression analysis to identify resilience factors within families by family parenting stress measured with the OBVL

					Change statistics				
	R	R^2^	Adjusted R^2^	SE of the estimate	R^2^ change	F change	df1	df2	Sig. F change
Family parenting stress + resilience factors	.70^a^	.49	.45	18.22	.49	11.88	5	61	< .01

	Unstandardized coefficients		Standardized coefficients	t	Sig	95.0% Confidence Interval for B	
	B	SE	Beta			Lower bound	Upper bound
(Constant)	188.04	20.53		9.15	< .01	146.97	229.10
Planning & organizing skills mothers	.22	.61	.03	.35	.72	− 1.00	1.45
Worrying mothers	.56	.49	.12	1.15	.25	− .41	1.55
Social relations mothers	− 3.26	1.15	− .34	− 2.81	< .01	− 5.58	− .94
Worrying fathers	.94	.59	.18	1.57	.12	− .25	2.13
Social relations fathers	− 1.92	.98	− .24	− 1.96	.05	− 3.88	.03

Dependent variable: family parenting stress

^a^Predictors: (constant), social relations fathers, worrying mothers, planning & organizing skills mothers, worrying fathers, social relations mothers

^b^Dependent variable: family parenting stress

## Discussion

The present study shows that within families of young children with ASD, the proportion of families in which both parents experience a desirable degree of parenting stress referred to in this article as regular parenting stress is almost equal (33%) to the percentage of families with both parents experiencing a degree of stress that needs attention for the wellbeing of the parent and the child, referred to in this article as high parenting stress (36%). Families with mothers experiencing high, and fathers with regular parenting stress, are twice as frequent (22%) as the other way around (9%). Good planning and organizing skills and satisfying social relations in mothers related to less maternal parenting stress. A positive view of the future and satisfactory social relations in fathers related to less paternal parenting stress. As worrying can lead to an overall negative mood and depression. The core contributing factor to resilience within families, i.e., less family parenting stress comprising out of the combined parenting stress of both mother and father from the same family, showed to be the satisfaction of both parents with their social relations. In summery, the individual dynamics of resilience factors in mothers and fathers contribute differently to their parenting stress. Within families both mothers and fathers satisfactory with their social relations is crucial to resilience within families.

New in the current study is that a within family approach was used by examining resilience to parenting stress in both mothers and fathers of the same young child with ASD, giving us insight into family dynamics of resilience to parenting stress. To our knowledge, this approach has not previously been used in scientific research within this population.

The majority (69%) of the parents of the same young child with ASD experience a comparable degree of parental stress i.e., both parents experience regular or both parents experience high parenting stress, instead of one parent reporting much more stress than the other within the same family. Similarities in parenting stress may be related to factors associated to the child and/or family relationships like the influence of the partners on each other in coping with stress (Bonis, 2016; Enea & Rusu, 2020; Ooi et al., 2016; Walsh, 2016). Another explanation for this could be that on average, members of romantic dyads tend to be more alike, according to the underlying theory of assortative mating. Assortative mating being a mating pattern in which individuals with similar phenotypes or genotypes mate with one another more frequently than would be expected under chance (Jiang et al., 2013). Although to our knowledge no research has been done on resilience within families to parenting stress and assortative mating, we can hypothesize that the explanation of this similarity between parents regarding parental stress could be based on this theory, although the influence of the partners on each other in coping with stress might also explain similarity in parental stress response.

The families in which mothers experience high and fathers regular parenting stress (22%) are twice as many as the families in which the experience of parenting stress for mothers and fathers is the other way around (9%). A possible explanation for this may be that intrapersonal factors associated to parenting stress at group level, differed for mothers and fathers. This is partly in line with previous robust literature on parental stress at group level, frequently showing that mothers of (young) children with ASD experience high parenting stress, as is the case within this study (Bonis, 2016; Dijkstra-de Neijs et al., 2020; Enea & Rusu, 2020; Estes et al., 2009; Ooi et al., 2016; Rezendes & Scarpa, 2011; Riahi & Izadi-Mazidi, 2012). The results within this study strengthen the literature on increased parenting stress also present in fathers, in which previous studies had varying results (Baker-Ericzén et al., 2005; Flippin & Crais, 2011; Grebe et al., 2021; Pisula & Porębowicz-Dörsmann, 2017; Rivard et al., 2014; Soltanifar et al., 2015). Although not investigated within this study, mothers are more often the primary care giver for the child and may thereby be more frequently exposed to stressful parenting situations in which their planning and organization skills are called upon than fathers. This gender specific pattern on the difference in perceived parenting stress by mothers and fathers is a known clinically observation but to our knowledge a novelty within the literature on resilience within families, already present in early parenthood, concerning parental intrapersonal resilience factors to parenting stress. However, this is an outcome of this study that concerns the entire study population and is not just about the 22% group.

Subsequently, this study explored which individual factors of mothers and fathers within the same family or of the same young child with ASD related to better resilience within families to family parenting stress. The present study shows that the satisfaction of both parents with their social relationships is the resilience factor to family parenting stress. The results of this study are partially in line with the universal findings at group level, comprising mostly research on mothers, that a satisfactory social network helps with raising their child (Ekas & Lickenbrock, 2010; Li et al., 2022; Siman-Tov & Kaniel, 2011; Tobing & Glenwick, 2007; Weinberg et al., 2021). This study adds that this is also the case for fathers making social support the main factor contributing to resilience within families of the same young child with ASD. Interventions for families with young children with ASD could pay extra attention to parents’ satisfaction with their social relations, so that these families are supported in increasing their resilience to parenting stress.

### Strengths and Limitations

We included a large group of individuals (n = 249), comprising mothers (n = 84) and fathers (n = 74) of young children with ASD (n = 88) of which 74 families comprised data on the mothers and fathers of the same young child with ASD. Specifically, we assessed not only the individual parent, both mothers and fathers, but also the combined resilience of the family, in which mothers and fathers of the same young child with ASD were incorporated. This contributes to the robustness of our findings and provides a more dynamic view on parenting stress in families with young children with ASD, concerning the entire family, including the well-being of the child(ren).

There are also limitations within this study as the current study used a cross-sectional design, thus changes over time could not be observed. Also, although the operationalization of resilience to parental stress has been carefully done, resilience is a very broad concept, so the measured variables within this study may not be all-encompassing. Additionally, this study examines resilience from a psychological perspective which leads to new insights into this concept but still leaves questions regarding the underlying mechanisms between resilience, parenting stress and the physical health of parents of young children with ASD, as within this group also high risks on stress related diseases are present (Dijkstra-de Neijs et al., 2020). Finally, we would like to add that families within this study were not asked their preferences for autism terminology. The choice to use the APA terminology (ASD) comes from the research team.

### Future Research

In addition, future research might benefit from a more comprehensive approach on factors contributing to resilience within families to parenting stress in families with young children with ASD. For instance, by including physical parameters, providing a more holistic insight into mental and physical stress and resilience factors.

## Conclusion

This study shows that there are differences in intrapersonal factors contributing to resilience when mothers and fathers raise their child with ASD. When addressing parenting stress in parents of young children with ASD, these differences should be considered. Higher satisfaction with social relationships by both mother and father of the same young child with ASD makes these families more resilient in coping with the daily parenting challenges they encounter, associating with lower levels of family parenting stress*.* Actively focusing on enhancing the satisfaction of the social relationships of both parents, might therefore benefit the health and development of the whole family.

## Data Availability

Not applicable.
